# Attitude of resident doctors towards intensive care units’ alarm settings

**DOI:** 10.4103/0019-5049.72640

**Published:** 2010

**Authors:** Rakesh Garg, Anju R Bhalotra, Nitesh Goel, Amit Pruthi, Poonam Bhadoria, Raktima Anand

**Affiliations:** Department of Anaesthesiology and Intensive Care, Maulana Azad Medical College, New Delhi, India

**Keywords:** Adjustment, alarms, critical care, intensive care units

## Abstract

Intensive care unit (ICU) monitors have alarm options to intimate the staff of critical incidents but these alarms needs to be adjusted in every patient. With this objective in mind, this study was done among resident doctors, with the aim of assessing the existing attitude among resident doctors towards ICU alarm settings. This study was conducted among residents working at ICU of a multispeciality centre, with the help of a printed questionnaire. The study involved 80 residents. All residents were in full agreement on routine use of ECG, pulse oximeter, capnograph and NIBP monitoring. 86% residents realised the necessity of monitoring oxygen concentration, apnoea monitoring and expired minute ventilation monitoring. 87% PGs and 70% SRs routinely checked alarm limits for various parameters. 50% PGs and 46.6% SRs set these alarm limits. The initial response to an alarm among all the residents was to disable the alarm temporarily and try to look for a cause. 92% of PGs and 98% of SRs were aware of alarms priority and colour coding. 55% residents believed that the alarm occurred due to patient disturbance, 15% believed that alarm was due to technical problem with monitor/sensor and 30% thought it was truly related to patient’s clinical status. 82% residents set the alarms by themselves, 10% believed that alarms should be adjusted by nurse, 4% believed the technical staff should take responsibility of setting alarm limits and 4% believed that alarm levels should be pre-adjusted by the manufacturer. We conclude that although alarms are an important, indispensable, and lifesaving feature, they can be a nuisance and can compromise quality and safety of care by frequent false positive alarms. We should be familiar of the alarm modes, check and reset the alarm settings at regular interval or after a change in clinical status of the patient.

## INTRODUCTION

The clinical environment contains a plethora of bells, beeps, and buzzers. Intensive care unit (ICU) monitors have alarm options to intimate the staff of critical incidents.[1] Since critical patients have different underlying diseases, the ability of ICU monitors to detect abnormality is limited and needs to be appropriately adjusted. With this objective in mind, this study was done among resident doctors, with the aim of assessing the existing attitude among resident doctors towards ICU alarm settings.

## METHODS

This study was conducted among residents working at ICU of a multispeciality centre, with the help of a printed questionnaire.

1. What all monitors are routinely employed in the ICU?
2. Are you aware of presence of types of alarm and settings of alarm limits on the monitor?
3. Do you check whether alarm limits have been set for various parameters?
4. Do you set the alarm limits daily for each and every parameter?
5. Within what range of the baseline do you set these limits?
6. What do you do when an alarm sounds in any of the monitors? (Choose to ignore it the first time it sounds, Disable it temporarily, Disable it temporarily and start looking for a cause yourself, Disable it temporarily and summon expert senior help)
7. What do you do in case the above measures fail and the alarm keeps on sounding persistently? (Increase alarm limits, Choose to ignore the alarm, Try to disable the alarm permanently, Switch off the monitor)
8. Are you aware of the alarms priority and colour coding of alarms?
9. What are the various reasons of alarms in ICU? (Truly related to patient clinical status, Technical error, Patient movement/during disturbance of sensor by doctor/nursing personnel)
10. Who should set the alarm levels?

The data were spread on excel sheet and analysed.

## RESULTS

The study involved 80 residents. Of these, 34 were postgraduate residents (PG) and the rest were senior residents (SRs) in the field of anaesthesiology. All residents were in full agreement on routine use of electro cardio gram (ECG), pulse oximeter, capnograph and non invasive blood pressure (NIBP) monitoring. 86% residents realised the necessity of monitoring oxygen concentration, apnoea monitoring and expired minute ventilation monitoring. This awareness was best among 1^st^ year postgraduates and 3^rd^ year SRs. 100% of residents were aware of presence of types of alarm and settings of alarm limits on the monitor. 87% PGs and 70% SRs routinely checked alarm limits for various parameters. 50% PGs and 46.6% SRs set these alarm limits daily for all parameters. Awareness increased from 1^st^ to 3^rd^ year PGs but decreased form 1^st^ to 3^rd^ year SRs. 11% of the residents set 30% as alarm limits, 10% residents preferred a 15% range, 3% residents set a limit of 10%, 3% residents set a limit of 25% and the rest set alarm limits of 20%. The initial response to an alarm among all the residents was to disable the alarm temporarily and try to look for a cause. 55% of PGs and 66% of SRs increased the alarm limits so that the alarm would stop sounding. If their attempts to find a cause becomes unsuccessful, 14% of 3^rd^ year SRs ignored the alarm altogether and 6% of 2^nd^ year PGs disabled the alarm permanently. 92% of PGs and 98% of SRs were aware of alarms priority and colour coding. 55% residents believed that the alarm occurred due to patient disturbance, 15% believed that alarm was due to technical problem with monitor/sensor and 30% thought it was truly related to patient’s clinical status. 82% residents set the alarms by themselves, 10% believed that alarms should be adjusted by nurse, 4% believed the technical staff should take responsibility of setting alarm limits and 4% believed that alarm levels should be pre-adjusted by the manufacturer.

## DISCUSSION

The need of various monitoring parameters varies among the patients because of their clinical status. In our study, we find that the residents were in full agreement regarding the need of basic monitoring parameters for patients in the ICU but varied response was observed regarding specific parameters and also for the alarm settings.

Many of the alarms are spurious due to patient movement, artefact, problems with the sensor, algorithms or the patient–equipment contact.[1] The proper settings of alarm limits are essential. The default alarm limits cannot be applicable for all patients because of the different baseline parameters and action required if the monitoring parameters change by a certain limit. Various studies have concluded that over 90% of alarm sounds may not be clinically important.[1,2] In a study, 72% of all alarms resulted in no medical action.[3] The study reported a positive and negative predictive value for alarms to be 27 and 99%, respectively. ICU alarms produce sound intensities above 80 decible (dB). False alarms in the ICU can lead to a disruption of care, impacting both the patient and the clinical staff through noise disturbances, desensitisation to warnings and slowing of response times, leading to decreased quality of care.[1] Also, sleep deprivation and depressed immune systems have been reported.[1] In a survey regarding ICU alarms, it was mentioned that 52.2% of the nurses considered themselves for controlling alarm limits.[4] Being able to set multiple levels with possibly different levels of alarm urgency would be a more ergonomic way of dealing with this problem. In all cases, it is necessary to set the threshold alarm limit. There is no standard for default alarm settings. Seibeg and others suggested combining of several alarms into a new one and the addition of trend alarms to allow narrower threshold alarm limits.[4]

In another clinical study, when asked for their opinion on how to adequately set monitoring alarms, cardiac anaesthesiologists named only heart rate and arterial blood pressure alarms, and all other cardiovascular alarms were disabled.[5] Regarding alarm limit settings, it was revealed that they would set systolic arterial pressure alarms at ±30 mmHg, heart rate alarms at ±30 bpm and the oxygen saturation lower alarm limit to 90%.[6] In an interventional study, there was a statistically significant improvement in the alarms’ reading as their limits were adjusted according to patient’s real value.[7]

We conclude that although alarms are an important, indispensable, and lifesaving feature, they can be a nuisance and can compromise quality and safety of care by frequent false positive alarms. We should be familiar of the alarm modes, check and reset the alarm settings at regular interval or after a change in clinical status of the patient.
